# Antimicrobial Resistance Pattern and Their Beta-Lactamase Encoding Genes among *Pseudomonas aeruginosa* Strains Isolated from Cancer Patients

**DOI:** 10.1155/2014/101635

**Published:** 2014-02-23

**Authors:** Mai M. Zafer, Mohamed H. Al-Agamy, Hadir A. El-Mahallawy, Magdy A. Amin, Mohammed Seif El-Din Ashour

**Affiliations:** ^1^Department of Microbiology and Immunology, Faculty of Pharmacy, Ahram Canadian University, Cairo, Egypt; ^2^Department of Pharmaceutics and Microbiology, College of Pharmacy, King Saud University, P.O. Box 2457, Riyadh 11451, Saudi Arabia; ^3^Department of Microbiology and Immunology, Faculty of Pharmacy, Al-Azhar University, Cairo, Egypt; ^4^Department of Clinical Pathology, National Cancer Institute, Cairo University, Cairo, Egypt; ^5^Department of Microbiology and Immunology, Faculty of Pharmacy, Cairo University, Cairo, Egypt; ^6^Department of Microbiology and Immunology, Faculty of Pharmacy, Modern Science and Arts University, 6th October City, Giza, Egypt

## Abstract

This study was designed to investigate the prevalence of metallo-**β**-lactamases (MBL) and extended-spectrum **β**-lactamases (ESBL) in *P. aeruginosa* isolates collected from two different hospitals in Cairo, Egypt. Antibiotic susceptibility testing and phenotypic screening for ESBLs and MBLs were performed on 122 *P. aeruginosa* isolates collected in the period from January 2011 to March 2012. MICs were determined. ESBLs and MBLs genes were sought by PCR. The resistant rate to imipenem was 39.34%. The resistance rates for *P. aeruginosa* to cefuroxime, cefoperazone, ceftazidime, aztreonam, and piperacillin/tazobactam were 87.7%, 80.3%, 60.6%, 45.1%, and 25.4%, respectively. Out of 122 *P. aeruginosa*, 27% and 7.4% were MBL and ESBL, respectively. The prevalence of *bla*_VIM**-**2_, *bla*_OXA**-**10_-, *bla*_VEB**-**1_, *bla*_NDM_-, and *bla*_IMP**-**1_-like genes were found in 58.3%, 41.7%, 10.4%, 4.2%, and 2.1%, respectively. GIM-, SPM-, SIM-, and OXA-2-like genes were not detected in this study. OXA-10-like gene was concomitant with VIM-2 and/or VEB. Twelve isolates harbored both OXA-10 and VIM-2; two isolates carried both OXA-10 and VEB. Only one strain contained OXA-10, VIM-2, and VEB. In conclusion, *bla*_VIM**-**2_- and *bla*_OXA**-**10_-like genes were the most prevalent genes in *P. aeruginosa* in Egypt. To our knowledge, this is the first report of *bla*_VIM**-**2_, *bla*_IMP**-**1_, *bla*_NDM_, and *bla*_OXA**-**10_ in *P. aeruginosa* in Egypt.

## 1. Introduction

*Pseudomonas aeruginosa* is widely known as an opportunistic organism, frequently involved in infections of immune-suppressed patients, and also causes outbreaks of hospital-acquired infections [1] that cause infections with a high mortality rate [2]. This latter is, in part, attributable to the organism's intrinsically high resistance to many antimicrobials and the development of increased, particularly multidrug, resistance in healthcare settings [3], both of which complicate antipseudomonal chemotherapy. The carbapenems have been the drug of choice for the treatment of infections caused by penicillin or cephalosporin resistant Gram-negative bacilli [4]. However, carbapenem resistance has been observed frequently in nonfermenting bacilli *Pseudomonas aeruginosa* and *Acinetobacter* spp. Resistance to carbapenem is due to decreased outer membrane permeability, increased efflux systems, alteration of penicillin-binding proteins, and carbapenem hydrolyzing enzymes-carbapenemase. In the last decade, several classes A, B, and D *β*-lactamases have been detected in *P. aeruginosa* [5]. The carbapenemases found are mostly metallo-*β*-lactamases (MBL), including IMP, VIM, SPM, SIM, GIM, AIM, DIM, or NDM enzymes, but serine carbapenemases have also been recorded, including KPC and GES variants [6].

The OXA-ESBLs are mutants of OXA-2 and -10, belonging to class D, whereas the other ESBL belongs to class A. VEB and PER types were found to be the most common (or least rare) ESBL in *P. aeruginosa* in several countries, contrasting to the dominance of CTX-M, SHV, and TEM ESBL in *Enterobacteriaceae* [7]. Detection of MBL and ESBL producing Gram-negative bacilli especially *P. aeruginosa* is crucial for optimal treatment of patients particularly critically ill and hospitalized patients and to control the spread of resistance. The aim of the present study was phenotypic and genotypic screening for MBL and ESBL producing strains among *P. aeruginosa* isolated from clinical specimens of cancer patients recovered from two hospitals in Cairo, Egypt.

## 2. Materials and Methods

### 2.1. Bacterial Strains

Hundred twenty-two nonduplicate nonconsecutive *P. aeruginosa* isolates were obtained from clinical specimens submitted for bacteriological testing from hospitalized in-patients admitted to Kasr El Aini Hospital and National Cancer Institute, Cairo University, Egypt, in the period from January 2011 to March 2012. Kasr El Aini School of Medicine and National Cancer Institute are tertiary hospitals belonging to Cairo University, Egypt. The study was approved by Ethics Committee of Cairo University and an informed consent was obtained from all patients receiving treatment and participating in the study. With regard to the specimen site, *P. aeruginosa* were isolated from wound swabs (*n* = 44), blood (*n* = 29), urine (*n* = 22), sputum (*n* = 11), cerebrospinal fluid (CSF) (*n* = 2), genital sites (*n* = 2), catheter tip (*n* = 2), central venous catheter (*n* = 4), ear swab (*n* = 2), pleural tissue specimen (*n* = 2), corneal graft (*n* = 1), and breast abscess (*n* = 1).

### 2.2. Bacterial Identification

Identification of *P. aeruginosa* was done on the basis of Gram staining, colony morphologies on MacConkey's agar, motility, pigment production, oxidase reaction, growth at 42°C, and the biochemical tests included in the API 20NE identification kit (Biomerieux, Marcy l'Étoile, France). The Vitek 2 system (Vitek 2 software, version R02.03; Advanced Expert System [AES] software, version R02.00N (bioMerieux, Marcy l'Étoile, France) was used with the ID-GNB card for identification of Gram-negative bacilli. The identified strains were stored in glycerol broth cultures at −70°C.

### 2.3. Antimicrobial Susceptibility Testing

Susceptibility of the isolates to the following antibacterial agents was tested by the Kirby-Bauer disc diffusion method [8] using disks (Oxoid ltd., Basin Stoke, Hants, England) on Mueller Hinton agar and interpreted as recommended by Clinical and Laboratory Standards Institute (CLSI) guidelines [9]: amikacin (AK, 30 *μ*g), aztreonam (ATM, 30 *μ*g), cefepime (FEP, 30 *μ*g), cefoperazone (CFP, 30 *μ*g), cefotaxime (CTX, 30 *μ*g), ceftazidime (CAZ, 30 *μ*g), ceftriaxone (CRO, 30 *μ*g), cefuroxime (CXM, 30 *μ*g), ciprofloxacin (CIP, 5 *μ*g), imipenem (IPM, 10 *μ*g), meropenem (MEM, 10 *μ*g), piperacillin/tazobactam (TPZ, 100/10 *μ*g), polymyxin B (PB, 300 units), and tobramycin (TOB, 10 *μ*g).

### 2.4. MIC Determination for MBL-Producing *P. aeruginosa*

The MICs of 9 antibiotics (cefepime, piperacillin/tazobactam, ceftazidime/clavulanic acid, ceftazidime, ciprofloxacin, amikacin, gentamicin, imipenem, and cefotaxime) were determined to 33 *P. aeruginosa* isolates that phenotypically produce MBL using Etest (AB Biodisk, Solna, Sweden) as described by the manufacturer. Results were interpreted using CLSI criteria for susceptibility testing [9]. *P. aeruginosa* ATCC 27853 was used as the reference strain.

### 2.5. Phenotypic Detection of ESBL

Combined double disc synergy test was performed with discs containing ceftazidime (30 *μ*g) alone and in the presence of clavulanate (10 *μ*g). In order to inhibit cephalosporinase overproduction, double disc synergy tests were also carried out with the addition of 400 *μ*g of boronic acid [10]. Increase in ceftazidime inhibition zone of ≥5 mm in the presence of clavulanic acid as compared with when tested alone was considered to be ESBL producer.

### 2.6. Phenotypic Detection of MBL

A 4 *μ*L of 0.5 M EDTA (Sigma Chemicals, St. Louis, MO) was poured on imipenem and ceftazidime disks to obtain a desired concentration of 750 *μ*g per disk. The EDTA impregnated antibiotic disks were dried immediately in an incubator and stored at −20°C in air-tight vials without desiccant until used. 0.5 McFarland equivalent overnight broth culture of test strain was inoculated on a plate of Mueller Hinton agar. One 10 *μ*g imipenem and one 30 *μ*g ceftazidime disks were placed on the agar plate. One each of EDTA impregnated imipenem and ceftazidime disks were also placed on same agar plate. The plate was incubated at 37°C for 16 to 18 h. An increase in the zone size of ≥7 mm around the imipenem-EDTA disk or ceftazidime-EDTA compared to imipenem or ceftazidime disks without EDTA was recorded as MBL producing strain [11].

### 2.7. Preparation of DNA Template for PCR

DNA templates were prepared according to the previous described method [12]. A 300 *μ*L of overnight culture of the test isolates in tryptone soy broth (Difco, Detroit, MI, USA) was centrifuged. The bacterial pellet was resuspended to the initial volume with HPLC grade water. The DNA template was prepared by boiling of suspension of bacterial pellet for 10 min and directly used in the PCR assay.

### 2.8. Detection of ESBL and MBL Genes

Genes for ESBLs (OXA-10-like gene, OXA-2-like gene, and VEB) and MBLs (VIM-1, VIM-2, IMP-1, IMP-2, SIM, GIM, SPM, and NDM) were sought by PCR for all isolates using the primers listed in Table 1 according to the previous protocols [7, 13–16]. Negative and positive controls were involved in all PCR experiments. Five *μ*L of reaction mix containing PCR product was analysed by electrophoresis in 0.8% (w/v) agarose (Fermentas, Lithuania).

## 3. Results

The antimicrobial susceptibility testing was done by disc diffusion method to 122 clinical isolates of *P. aeruginosa* that were collected from Kasr El Aini Hospital and National Cancer Institute, Cairo University, Egypt, in the period from January 2011 to March 2012.

The resistant rates of antibiotics are shown in Table 2. Forty-eight (39.34%) out of 122* P. aeruginosa* isolates were resistant to imipenem. Eight (6.5%) out of 122 isolates of *P. aeruginosa* showed intermediate resistance to imipenem. Fifty-six (46%) out of 122 *P. aeruginosa* isolates were resistant to meropenem. Only two isolates (1.64%) out of 122 showed intermediate resistance to meropenem. The resistant rates for *β*-lactam antibiotics including cefuroxime, cefoperazone, ceftazidime, aztreonam, and piperacillin/tazobactam were 87.7%, 80.3%, 60.6%, 45.1%, and 25.4%, respectively. The resistant rates for non-*β*-lactam antibiotics including gentamicin, ciprofloxacin, and amikacin were 50%, 43.4%, and 32.8%, respectively. Only 3 (2.5%) out of 122 *P. aeruginosa* isolates were resistant to polymyxin B. The antimicrobial resistance rates were higher for imipenem-resistant than imipenem-susceptible *P. aeruginosa* isolates (Table 2). Non-*β*-lactams showed higher activity against imipenem-resistant *P. aeruginosa* than *β*-lactams.

Use of combined disk method (imipenem, ceftazidime/imipenem, and ceftazidime + EDTA) for phenotypic production of MBL allowed the detection of 33 of 122 (27%) *P. aeruginosa* isolates.

Combined double disc synergy test was applied to detect ESBL in 122 *P. aeruginosa* isolates using ceftazidime alone or with clavulanic acid. Of these, only 9 (7.4%) isolates were positive for production of ESBL. All ESBL-producing *P. aeruginosa* were found to be resistant to ceftazidime. Five out of 33 MBL-producing isolates were found to produce ESBL and MBL simultaneously.

The results of MICs for MBL-producing *P. aeruginosa* isolates appeared in Table 3 which showed that the strains have high resistance toward imipenem, cefotaxime, gentamicin, and ciprofloxacin as their MIC was above the break points recommended by CLSI. The effect of clavulanic acid on the susceptibility was found as some isolates showed a decrease in MIC more than 3 doubling dilutions, and that indicates the presence of ESBL. On the other hand, according to the breakpoints recommended by CLSI, more than half of the isolates were sensitive toward piperacillin/tazobactam, ceftazidime, and cefepime and ceftazidime/clavulanic acid.

PCR experiments revealed amplification of 865 bp fragment corresponding to *bla*_VIM-2_-like gene in 28 of 48 (58.3%) imipenem-resistant isolates and a 760 bp fragment corresponding to *bla*_OXA-10_-like gene in 20 (41.5%) of imipenem-resistant isolates and a 642 bp fragment corresponding to *bla*_VEB_ gene in 5 (10.4%) of isolates; two isolates (4.2%) showed a fragment of 984 bp corresponding to *bla*_NDM_ gene and only one isolate (2.1%) showed a fragment of a 740 bp corresponding to *bla*_IMP-1_. In this work MBL *bla*_VIM-1_, *bla*_IMP-2_, *bla*_GIM_, *bla*_SIM_, and *bla*_SPM_ allele were not detected; also OXA-2 like gene was not detected in this study.

OXA-10-like gene was concomitant with VIM-2 and/or VEB. Twelve isolates harbored both OXA-10 and VIM-2; however two isolates carried both OXA-10 and VEB. Only one MBL-producing strain contained OXA-10, VIM-2, and VEB (Table 4).

## 4. Discussion

Carbapenems are among the best choices for the treatment of infections caused by multidrug resistant Gram-negative rods. In recent years, Egypt has been considered among the countries that reported high rates of antimicrobial resistance [17]. In the present study, there were high levels of resistance to all commercially available antimicrobial agents among *P. aeruginosa* isolated from Kasr El Aini Hospital and National Cancer Institute, Cairo University, Egypt; the rate of 39.3% imipenem-resistant isolates, this rate of carbapenem resistance reflects a threat limiting the treatment options in our hospitals. This can be explained in part by the increase in consumption of antimicrobial agents in the last decade leading to a selective pressure of antibiotics on *P. aeruginosa* and consequently the bacteria modify the resistant mechanisms. A similar high rate of resistance has been reported in many developing countries worldwide [18]. In Egypt, Ashour and El-Sharif [19] concluded that *Acinetobacter* and *Pseudomonas* species exhibited the highest resistance levels to imipenem (37.03%) among other Gram-negative organisms [19]. Also Mahmoud et al. [17] showed that among *P. aeruginosa* strains 33.3% were resistant to imipenem [17].

In the Middle East the occurrence of imipenem resistant *P. aeruginosa* is alarmingly recognized. In Saudi Arabia, the resistance rate of *P. aeruginosa* to imipenem was increased to 38.57% in 2011 [20]. Among 33 European countries participating in the European Antimicrobial Resistance Surveillance System in 2007, six countries reported carbapenem resistance rates of >25% among *P. aeruginosa* isolates; the highest rate was reported from Greece (51%) [21].

The clinically important MBL families are located in horizontally transferrable gene cassettes and can be spread among Gram-negative bacteria. Although we have not studied this horizontal transfer in the current study, it has been well demonstrated by several previous reports from other groups. Different families of these enzymes have been reported from several geographical regions so far. The most commonly reported families are IMP (for active on imipenem, first isolated in Japan), VIM (for Verona Integron-encoded metallo-*β*-lactamase, first isolated in Italy), GIM (for German Imipenemase), SPM (for Sao Paulo metallo-*β*-lactamase, first isolated in Brazil), and SIM (for Seoul Imipenemase, first isolated in Korea). IMP- and VIM-producing *Pseudomonas* strains have been reported worldwide, in different geographical areas [22]. In the current study 27% of 122 total *P. aeruginosa* isolates were positive for the production of MBL based on the results of phenotypic screening for MBL. This was lower than the prevalence of MBL producers in Egyptian study which was 32.3% [23]. However our finding agreed with an Indian study in which 28.57% of *P. aeruginosa* was found to produce MBL [24]. In the present study, VIM-2 was the most frequently detectable gene among the different MBL genes investigated; the percent of 58.3% among imipenem-resistant *P. aeruginosa* was detected.

This finding was supported by results of previous studies demonstrating VIM-2 as the most dominant MBL implicated in imipenem resistant *P. aeruginosa* and confers the greatest clinical threat [25]. Worldwide, VIM-2 is the dominant MBL gene associated with nosocomial outbreaks due to MBL-producing *P. aeruginosa* [26]. In our study, out of 33 (27%) MBL producers, 26 (78.8%) were positive for genes detected by PCR and 15 (31.3%) out of 48 imipenem resistant isolates were positive for genes investigated by PCR and in the same time were negative MBL producers. This indicated that there are other resistance mechanisms to carbapenem such as class A carbapenemases including KPC and GES variants and MBLs were not the sole mechanism of carbapenem resistance in the present study. The imipenem resistant strain with no phenotypic or genotypic sign of MBL production may possess other enzyme mediating carbapenem resistance such as AmpC beta lactamase and/or other mechanisms such as membrane permeability and efflux mechanisms.

Class A ESBLs are typically identified in *P. aeruginosa* isolates showing resistance to extended-spectrum cephalosporin (ESCs) [27]. Classical ESBLs have evolved from restricted-spectrum class A TEM and SHV *β*-lactamases although a variety of non-TEM and non-SHV class A ESBLs have been described such as CTX-M, PER, VEB, GES, and BEL [5] and class D ESBLs derived from narrow-spectrum OXA *β*-lactamases are also well known [28]. Structural genes VEB, OXA, and PER types are the most common ESBLs reported in *P. aeruginosa* [7].

In the present study, production of ESBL was detected in only (7.4%) out of 122 *P. aeruginosa* isolates. This was much lower than what was found in a study done by Gharib et al. in 2009 in Egypt in which 24.5% were ESBL producers [29]. In the present study high prevalence of *bla*_OXA-10_ was detected in imipenem resistant *P. aeruginosa* isolates; twenty of 48 (41.7%) isolates resistant to imipenem were OXA-10 positive followed by VEB-1 which was detected in 5 (10.4%). In a recent study in Iran, most prevalent ESBL genes included OXA-10 (70%) and PER-1 (50%) followed by VEB-1 (31.3%) [30]. This study agreed with our study in the prevalence of OXA-10 in ESBL-producing *P. aeruginosa.* However VEB type ESBLs were the predominant ESBL reported in *P. aeruginosa* in a number of studies where ESBLs were commonly seen [7, 31]. Phenotypic methods for detection of ESBL are not reliable in *P. aeruginosa* strains and PCR is advisable since only 9 isolates were ESBL producers upon phenotypic screening while 20 isolates were positive OXA-10 and 5 were VEB positive using PCR for their detection.

KPC rarely was detected in *P. aeruginosa*; however the number of reports of KPC-producing *P. aeruginosa* is increasing [32]. In this study, we did not test KPC. KPC and another rarely carbapenemases may be found in ESBL-producing strains because most of them had reduced susceptibility to imipenem (MIC 2–8 mg/L).

In the current study 97.5% of the total *P. aeruginosa* isolates were sensitive to polymyxin B. This supports the evidence that polymyxin B has increasingly become the last viable therapeutic option for multidrug resistant (MDR) *Pseudomonas* infections. This result agreed with a study done by Tawfik el al. in 2012 which they found that all isolates were sensitive to polymyxin [33].

In conclusion, the rates of MBL-producing *P. aeruginosa* and ESBL-producing *P. aeruginosa* isolates from Kasr El Aini Hospital and National Cancer Institute, Cairo University, in Egypt were notable and, unfortunately, only a limited number of antimicrobial drugs are active. Therefore, MBL and ESBL screening should be implemented for routine laboratory studies in routine practice. VIM-2 is the most prevalent MBL producing *P. aeruginosa* in Egypt. OXA-10 is the most prevalent ESBL producing *P. aeruginosa* in Egypt. MBL is much more prevalent than ESBL as mechanism of resistance in *P. aeruginosa*. Molecular techniques are more reliable than phenotypic screening in detecting ESBL production in *P. aeruginosa* strains. Further studies are needed to specify the most important genes of resistance among *P. aeruginosa* in Egypt.

## Figures and Tables

**Table 1:** Primers used for detection of MBL, OXA 10, and VEB.

Primers	Sequence	Reference	Expected PCR product
*bl* *a*_IMP-1_	TGAGCAAGTTATCTGTATTC TTAGTTGCTTGGTTTTGATG	[14]	740 bp

*bl* *a*_IMP-2_	GGCAGTCGCCCTAAAACAAA TAGTTACTTGGCTGTGATGG	[14]	737 bp

*bl* *a*_VIM-1_	TTATGGAGCAGCAACGATGT CAAAAGTCCCGCTCCAACGA	[14]	920 bp

*bl* *a*_VIM-2_	AAAGTTATGCCGCACTCACC TGCAACTTCATGTTATGCCG	[14]	865 bp

*bl* *a*_NDM_	CACCTCATGTTTGAATTCGCC CTCTGTCACATCGAAATCGC	[16]	984 bp

*bl* *a*_OXA-10_	TATCGCGTGTCTTTCGAGTA TTAGCCACCAATGATGCCC	[7]	760 bp

*bl* *a*_VEB-1_	CGACTTCCATTTCCCGATGC GGACTCTGCAACAAATACGC	[7]	642 bp

*bl* *a*_OXA-2_	GCCAAAGGCACGATAGTTGT GCGTCCGAGTTGACTGCCGG	[13]	700 bp

*bl* *a*_GIM_	TCGACACACCTT GGT CTG AA AACTTCCAACTT TGCCATGC	[15]	477 bp

*bl* *a*_SPM_	AAAATCTGGGTACGCAAA CG ACATTATCCGCTGGAACAGG	[15]	271 bp

*bl* *a*_SIM_	TAC AAG GGATTCGGCATCG TAATGG CCTGT CCCATG TG	[15]	570 bp

**Table 2:** Resistance rates for clinical *P.  aeruginosa* isolates.

Antibiotic	Number (%) of resistant isolates			
	Total isolates (*n* = 122)	Imipenem susceptible (*n* = 66)	Imipenem intermediate (*n* = 8)	Imipenem resistant (*n* = 48)
*β*-lactams				
Cefuroxime	107 (87.7%)	51 (77.2%)	8 (100%)	48 (100%)
Cefoperazone	98 (80.3%)	43 (65.2%)	8 (100%)	47 (97.9%)
Ceftazidime	74 (60.6%)	23 (34.8%)	8 (100%)	43 (89.5%)
Meropenem	56 (45.9%)	3 (4.5%)	7 (87.5%)	46 (95.8%)
Aztreonam	55 (45.1%)	28 (42.4%)	3 (37.5%)	24 (50%)
Imipenem	48 (39.3%)	66 (100%)	8 (100%)	48 (100%)
Piperacillin/tazobactam	31 (25.4%)	4 (6.1%)	7 (87.5%)	20 (41.6%)
Non *β*-lactams				
Gentamicin	61 (50%)	13 (19.7%)	5 (62.5%)	43 (89.5%)
Ciprofloxacin	53 (43.4%)	17 (25.8%)	5 (62.5%)	31 (64.5%)
Amikacin	40 (32.8%)	9 (13.6%)	7 (87.5%)	24 (50%)
Polymyxin B	3 (2.4%)	0 (0.0%)	1 (12.5%)	2 (4.2%)

**Table 3:** MICs of antibiotics for MBL-producing *P.  aeruginosa* isolates.

Isolates number	MICs (*μ*g/mL)								
	PM	PTc	TZ	TZL	CI	AK	GM	IP	CT
	≥32	≥128/4	≥128/2	≥32	≥4	≥64	≥4	≥16	≥32
1	≥256	16	≥256	≥256	2	12	3	≥32	≥256
2	12	64	8	3	≥32	16	≥256	≥32	≥256
3	8	64	64	24	≥32	≥256	12	≥32	≥256
4	4	3	6	2	≥32	≥256	≥256	≥32	32
5	4	3	6	1.5	≥32	≥256	≥256	≥32	32
6	64	≥256	≥256	≥256	≥32	≥256	≥256	≥32	≥256
7	24	≥256	≥256	192	≥32	≥256	12	≥32	≥256
8	6	96	96	24	0.094	4	2	≥32	≥256
9	≥256	3	≥256	≥256	≥32	32	12	≥32	≥256
10	≥256	2	4	1.5	≥32	2	2	≥32	16
11	≥256	≥256	≥256	≥256	≥32	≥256	32	≥32	≥256
12	6	24	8	16	≥32	≥256	1.5	≥32	≥256
13	≥256	≥256	≥256	≥256	≥32	≥256	≥256	≥32	≥256
14	≥256	24	≥256	≥256	1	6	2	≥32	≥256
15	16	24	16	4	≥32	12	≥256	≥32	≥256
16	≥256	≥256	48	8	≥32	96	≥256	≥32	≥256
17	6	12	12	2	≥32	6	≥256	≥32	≥256
18	≥256	4	≥256	≥256	≥32	96	32	≥32	≥256
19	64	≥256	≥256	96	0.094	96	32	≥32	≥256
20	8	32	12	2	≥32	16	≥256	≥32	≥256
21	16	16	24	4	≥32	48	≥256	≥32	≥256
22	256	≥256	≥256	≥256	≥32	128	32	≥32	≥256
23	≥256	≥256	≥56	256	≥32	≥256	32	≥32	≥256
24	8	12	≥256	4	0.064	8	≥256	≥32	≥256
25	≥256	≥256	≥256	128	≥32	48	32	≥32	≥256
26	≥256	≥256	≥256	≥256	≥32	≥256	≥256	≥32	≥256
27	16	≥256	≥256	32	0.064	8	32	≥32	≥256
28	64	≥256	≥256	≥256	0.064	32	32	≥32	≥256
29	2	4	12	2	0.125	4	2	≥32	≥256
30	2	4	8	1.5	0.125	4	2	≥32	≥256
31	8	128	6	24	0.094	6	2	≥32	≥256
32	12	32	2	8	0.064	6	2	≥32	≥256
33	6	≥256	8	3	≥32	128	2	≥32	≥256

PM: cefepime, PTc: piperacillin/tazobactam, TZL: ceftazidime/clavulanic acid, TZ: Ceftazidime, CI: ciprofloxacin, AK: Amikacin, GM: Gentamicin, IP: Imipenem, CT: Cefotaxime.

**Table 4:** Differential relation between genes investigated and phenotypic methods for MBL and ESBL producing *P.  aeruginosa*.

Genes investigated	VIM-2 (*n* = 28)	OXA-10 (*n* = 20)	VEB (*n* = 5)	NDM (*n* = 2)	IMP-1 (*n* = 1)
MBL (*n* = 33)					
Pos.	21	14	4	0	1
Neg.	7	6	1	2	0
ESBL (*n* = 9)					
Pos.	4	4	1	2	0
Neg.	24	16	4	0	1
VIM-2					
Pos.	28	12	2	0	1
Neg.	0	8	3	2	0
OXA-10					
Pos.	12	20	2	1	0
Neg.	16	0	3	1	1
VEB					
Pos.	2	2	5	0	0
Neg.	26	18	0	2	1
NDM					
Pos.	0	1	0	2	0
Neg.	28	19	5	0	1
IMP-1					
Pos.	1	0	0	0	1
Neg.	27	20	5	2	0
